# Unmet need and psychological distress predict emergency department visits in community-dwelling elderly women: a prospective cohort study

**DOI:** 10.1186/1471-2318-11-86

**Published:** 2011-12-19

**Authors:** Jacqueline M Quail, Christina Wolfson, Abby Lippman

**Affiliations:** 1 Department of Epidemiology and Biostatistics and Occupational Health, McGill University, Montreal, Quebec, Canada; 2 Research Institute, McGill University Health Centre, Montreal, Quebec, Canada; 3 Saskatchewan Health Quality Council, Saskatoon, Saskatchewan, Canada

## Abstract

**Background:**

Unmet need to perform activities of daily living (ADL) is associated with increased use of urgent health services by the elderly. However, the reported associations may be confounded by psychological distress. We examine the independent effects of unmet need and psychological distress upon emergency department (ED) visits.

**Methods:**

We conducted a prospective study of randomly selected community-dwelling adults aged ≥ 75. We report here the results for women only (n = 530). In-person interviews collected data on self-reported unmet need and the 14-item *l'Indice de détresse psychologique de Santé Québec *psychological distress scale. ED visits were identified from an administrative database. Multivariable logistic regression was used to identify predictors of any ED visit in the 6 months following the baseline interview.

**Results:**

In multivariable analysis, unmet need in instrumental ADL was associated with subsequent ED visits (odds ratio = 1.57, 95% confidence interval = 1.02-2.41), as was psychological distress (odds rate = 1.30, 95% confidence interval = 1.02-1.67). The magnitude of the association between unmet need and ED visits was overestimated in statistical models that did not adjust for psychological distress.

**Conclusions:**

Both unmet need and psychological distress were independent predictors of ED visits. Future investigations of unmet need and health services utilization should include psychological distress to control for confounding and improve the internal validity of statistical models.

## Background

In Canada, people age 65 and older comprise 13% of the population and it is estimated that this will rise to over 23% by the year 2031 [1]. As individuals age, they may experience worsening health and difficulty performing instrumental activities of daily living (IADL) or personal activities of daily living (PADL). IADL are necessary to maintain a household and function in the community; PADL are essential for self-care in everyday life [2]. Increasing age may lead to disability and the need for assistance to complete ADL, however, this need may not be met.

Allen and Mor (1997) described unmet need as the "perceived [inadequacy] of help received with activities the individual has difficulty performing or is unable to perform alone." The prevalence of unmet need varies according to the definition used, the population studied, and the specific ADL being examined [3-7]. In the community-dwelling disabled population, prevalence estimates of unmet need for PADL range from 11.7% for indoor ambulation to 22.6% for bathing; for IADL, they range from 14.9% for meal preparation to 25.8% for transportation to places beyond walking distance from the home [3]. Clearly unmet need is a common problem among disabled elderly persons.

Unmet need is associated with a variety of negative consequences that can affect the health and well-being of the disabled elderly. These range from feeling distressed because housework is not done to more serious experiences such as being unable to eat or drink when hungry or thirsty [3,8-10]. Allen and Mor (1997) found that nearly one-quarter (24.5%) of study participants who were disabled in transfers (i.e., moving from a chair to a bed, or from a chair to a wheelchair) had fallen, while 14.2% of those disabled in meal preparation reported they had been unable to eat when hungry. Many elderly people with unmet need continue to perform the tasks for which they need assistance despite both the difficulty of doing so and the potential for self-injury [3]. Given these potential negative consequences, it is not surprising that unmet need was associated with the increased use of health services in the three studies investigating this topic [3,10,11].

Allen and Mor (1997) found that community-dwelling disabled adults age 18 and older with any unmet PADL need reported more physician visits, emergency department visits, and hospitalizations than did those with all of their PADL needs met. However, the cross-sectional study design makes it impossible to know if unmet need preceded these events or vice versa. Additionally, Allen and Mor estimated only the crude association of unmet need with the use of these health services and so the findings may be confounded by other factors.

The two other studies expand upon Allen and Mor's work by using a prospective study design. Sands et al. (2006) studied community-dwelling PADL-disabled Americans aged 75 and older. They reported that after adjustment for important factors, those for whom all PADL needs were unmet were significantly more likely to have been hospitalized in the previous six months than those who had at least one of their PADL needs met [Odds Ratio (OR) = 1.26 (95% CI: 1.01-1.57)]. Once these individual's needs became met, however, their likelihood of being hospitalized decreased over the subsequent 12 weeks. Sands et al. limited their study population to only the most disabled seniors (i.e., those disabled in PADL) and so the generalizability of their findings to elderly persons who are less disabled who constitute a greater proportion of seniors living in the community is not known. Kuzuya et al. (2008) also prospectively investigated the effect of unmet need in one IADL, specifically medication management, among disabled community-dwelling elderly adults living in Nagoya, Japan. These researchers found that individuals with self-reported unmet need in medication management had an increased likelihood of being hospitalized, but not dying, in the subsequent three years.

Unfortunately, none of these studies considered the role of psychological distress, although Sands et al. investigated depression but did not include it in multivariable modelling because it was not statistically significantly associated with hospitalization. Unmet need is associated with concurrent depression [3,7], but depression is only one possible psychological response to, or cause of, unmet need. Anxiety, another relevant feature, may also be important, particularly if seniors are fearful of falling when performing difficult tasks or are frightened about the possibility of placement in a nursing home if they express a need for help. Investigation of psychological factors in relation to unmet need should be broadened to examine psychological distress, which is also associated with increased use of health services. Psychological distress is conceptualized as having four components [12]. In addition to anxiety and depression, psychological distress can manifest as irritability as well as cognitive problems that impede a person's judgement and ability to think clearly, such that a person has difficulty following simple instructions, misinterprets obvious information clues, or has difficulty remembering information and facts that are actually known well.

Previous research indicates that disability can lead to the development of one component of psychological distress, depression [13,14]. There is also evidence that depression can contribute to the development of disability through impaired ability and/or willingness to maintain one's health through proper nutrition, physical activity, and socialization [15,16]. This lack of self-care may extend to the development of unmet need. It is possible that the association between unmet need and psychological distress is bidirectional, in that disabled elderly persons with unmet need may become distressed as a result of the daily and difficult struggle to perform ADL, while psychological distress may prevent disabled elderly persons from actively seeking help and/or being able to accept help when it is offered. Over time, the reciprocal effect of one upon the other could lead to a mutually reinforcing cycle.

Andersen (1995) proposed a behavioural model of need, enabling, and predisposing factors that influence the use of health services [17,18]. Need factors relate to the medical needs that result from trauma, chronic disease, and disability. Enabling factors are those that influence access to health services such as availability of services, or travel and wait times. Predisposing factors are those that influence whether and to what extent a person will use health services and include age, sex, ethnicity, education, and psychological factors. Several studies of adults of all ages have shown that psychological distress can affect perceptions of health as well as health-seeking behaviours, including increased visits to a physician's office [19,20] or to an emergency department (ED) [21-23]. As a result, psychological distress may modify or confound the relationship between unmet need and health services use. We designed our research to explore this possibility by examining the effects of unmet need and psychological distress upon emergency department visits in a sample of community-dwelling elderly women. We initially set out to explore this association in both men and women. Because of the differences between men and women in the final years of their lives, we stratified our analyses by sex. The traditional role of men of an earlier generation was that of breadwinner and handyman; women's role included being housekeepers and homemakers. Consequently, many elderly men are either unwilling or unable to perform housekeeping and meal preparation. These tasks are usually performed by their wives. Moreover, men are more likely to have a chronic disease associated with high mortality than are women [24]. As their health and functional ability worsen, their needs are usually met by the care their wives provide. Women typically survive their husbands and end up living alone with limited financial resources [25]. Thus, older women are more 'exposed' to the possibility of unmet need. When we conducted the stratified analysis there were too few men with unmet need to be able to conduct an informative analysis and so we report here our findings for women only.

Research on unmet need has so far focused exclusively on disabled individuals since it is not possible to have unmet need without disability. This only allows an understanding of unmet need in reference to met need. We do not yet understand how individuals with unmet or met need differ from non-disabled individuals with regards to health outcomes. For this reason, we included non-disabled women in our research so that we could simultaneously examine the association of both met and unmet need in conjunction with psychological distress upon ED visits.

Based on our literature review, we know that both unmet need and psychological distress are independent predictors of ED visits, and that unmet need is likely to be associated with psychological distress. Thus, our objective is to elucidate the association of unmet need in conjunction with psychological distress upon ED visits. We hypothesize that unmet need, but not met need, is associated with an increased risk of visiting the ED. We further hypothesize that the association will be stronger for PADL unmet need than for IADL unmet need because disability in PADL is more severe. Finally, we hypothesize that psychological distress confounds and/or interacts with unmet need to increase the likelihood of visiting the ED. It is important to determine the effects of both of these factors upon the use of health care resources, especially as the elderly population continues to grow and an already strained health care system experiences increasing demand.

## Methods

### Source of Data

We analyzed data from the *Montreal Unmet Needs Study (MUNS)*, a prospective population-based cohort study of seniors living in Montreal, Quebec, Canada [26]. To be eligible to participate in the MUNS, individuals had to be 75 years of age or older, living in the community (i.e., not be institutionalized), able to speak English or French, and have no more than mild cognitive impairment as determined by a score of 14 or more on the telephone administered cognitive-screening *Adults Lifestyles & Function Interview (*ALFI). The ALFI is a telephone-administered scale that assesses cognitive impairment and has been shown to yield equivalent results to the face-to-face interview version of the *Mini Mental State Exam *(MMSE), with the exception of individuals with hearing impairments tending to score one point lower on average [27]. The MMSE has acceptable intra-tester reliability (r = 0.89) and inter-tester reliability (r = 0.83) and has demonstrated validity in geriatric populations [28]. The ALFI score ranges from 0 to 22 where a score of 17 or higher indicates no cognitive impairment, a score or 14 to 16 indicates mild cognitive impairment, and lower scores indicate progressively worse cognitive impairment. Potential participants were identified from a population-based survey list maintained by *Léger Marketing*, a marketing research firm that uses random digit dialling to conduct regular weekly telephone surveys of the Quebec population [29]. As part of these surveys, *Léger Marketing *records the age of respondents in categories, the oldest of which is "65 and older." From this survey list, *Léger Marketing *recruiters phoned 4,775 households known to contain a person 65 or older, were able to contact 4,420, and within these households identified 1300 seniors aged 75 and older who met the eligibility criteria. Of these, 946 (72.8%) agreed to be contacted by the research team. *Léger Marketing *forwarded a list of names of the eligible and orally consenting individuals to the MUNS coordinator who telephoned them to arrange a time to obtain informed consent and conduct the in-person interview. One hundred and seven potential participants changed their mind or could not be contacted, resulting in 839 (64.5%) individuals - 576 women and 263 men - who were interviewed face-to-face by trained study interviewers between February 2001 and March 2002.

During the interview, participants were also asked for permission for the researchers to obtain information pertaining to them contained in the government-managed *Régie de l'Assurance Maladie du Quebec *(RAMQ) prescription drug and medical services databases, and in the *Maintenance et Exploitation des Données pour l'Étude de la Clientèle Hospitalière *(MEDECHO) hospitalization database. Five hundred and thirty women (92.0%) and 253 men (96.2%) gave permission for linkage. Interview data were validated by two independent researchers and, as needed, study participants were contacted by telephone to clarify missing or contradictory information. As a result, fewer than 0.1% of the data are missing. The MUNS received approval from the *Ethics Committee of the Jewish General Hospital, l'Institut universitaire de gériatrie de Montreal *and the *McGill University Institutional Review Board*.

### Measurement

#### Disability and unmet need

**Figure 1:**
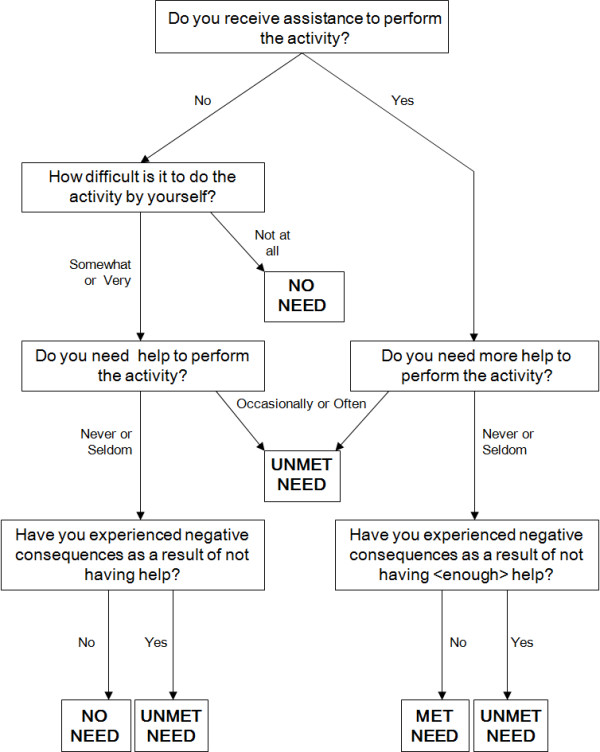
**Modified Algorithm to Determine Need for Assistance (Adapted from Allen and Mor, 1997)**.

The need for physical assistance to perform an activity of daily living (ADL) and whether that need was met or unmet were determined using a modified form of an algorithm created by Allen and Mor (1997) (Figure 1). The algorithm simultaneously determines a person's ability to perform specific daily activities and the perceived adequacy of any assistance received from another person. We modified the algorithm so that an individual who experiences difficulty when performing a PADL or IADL but reports having no need was categorized as "no need." This contrasts with the original Allen and Mor algorithm which categorizes such individuals as having "met need." Our modification seemed appropriate because it is not possible to have met need when no assistance is received.

MUNS participants were first asked about their need for physical assistance from another person to complete six personal activities of daily living (PADL) that are essential to daily life (dressing, bathing, indoor mobility, transfers, toileting, and feeding) and three instrumental activities of daily living (IADL) required to function in the community (meal preparation, housekeeping, and transportation to places beyond walking distance from the home). Study participants who reported having a need for physical assistance to complete any of these nine tasks were identified as being disabled. For every IADL or PADL in which a person was disabled, they were further identified as having met or unmet need. Using the information collected and applying the algorithm, participants were identified as having unmet need in a PADL or an IADL if they reported:

needing help but not receiving any help; or

receiving help, but needing more help; or

experiencing a negative consequence of not having any, or enough, help. Negative consequences include falling, having to wear dirty clothing, or being unable to eat or drink when hungry or thirsty.

Once each participant's level of need (no need, met need, or unmet need) was determined for each PADL and IADL, we aggregated the results into counts of met and unmet PADL and IADL need. We categorized IADL unmet need and IADL met need as 0, 1, and 2 or more, and PADL unmet need and PADL met need as 0, and 1 or more. A common way to model disability in statistical analyses is as a count of the ADL in which study participants are disabled. We used this approach, but took it a step further by subdividing disability into its four constituent components; that is, counts of IADL unmet need, IADL met need, PADL unmet need, and PADL met need. Modelling IADL met and unmet need as ordinal variables, and PADL met and unmet need as categorical variables, when taken together, represent the total number of ADL in which a person requires physical assistance (i.e., total disability). For example, a woman who is disabled in 5 ADL could have her information modeled in the following way: 2 IADL unmet needs, 1 IADL met need, 2 PADL unmet needs, and 0 PADL met needs. With this approach, we can adjust for disability (2 + 1 + 2 + 0 = 5) while separating out the individual effects of IADL and PADL met and unmet needs with ED visits.

#### Psychological Distress

**Figure 2:**
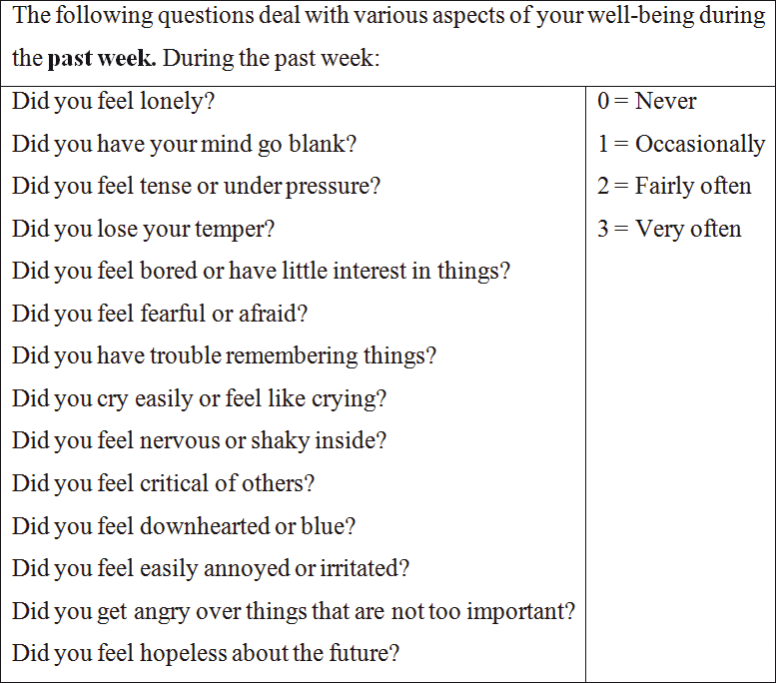
**l'Indice de détresse psychologique de Santé Québec**.

The official language of Montreal is French although a substantial proportion of Montreal residents speak English and so we chose a measure of psychological distress, *l'Indice de détresse psychologique de Santé Québec *(IDPESQ-14), that is available in both languages (Figure 2) The IDPESQ-14 has been validated in both French and English for construct, criterion, and predictive validity [30]. The IDPESQ-14 scale assesses the frequency of specific feelings and symptoms of psychological distress over the past week. That is to say, the occurrence of feelings of depression, anxiety, irritability, and impaired cognition. Responses to each of the 14 questions are limited to one of four categories with a numerical value assigned to each response ("never" = 0, "occasionally" = 1, "fairly often" = 2 and "very often" = 3). Response scores are summed, divided by the maximum score possible (42), and multiplied by 100 to create a 0 to 100 point scale: higher scores indicate higher levels of psychological distress. We transformed the raw ordinal scores into a linear interval-level scale using the Rasch measurement model [31], an established method to construct interval-level scales from ordinal scales [32-35]. The transformed psychological distress scores ranged from -4.6 to 2.7, where a higher score indicates more severe psychological distress.

#### Emergency department (ED) visits

All residents of Canada enjoy a universal health care system. Individuals can seek medical help at an ED for any reason at any time and the costs are fully covered by the government. We ascertained emergency department visits in the six months following the baseline interview from the RAMQ medical services database. We chose this follow-up period, rather than a longer one, to reduce the likelihood that a participant's need status may change between the baseline interview and subsequent ED visits which would introduce misclassification bias.

#### Covariates

Based upon Andersen's model, we included covariates in our analyses that may affect the association between psychological distress, unmet need, and ED visits. Predisposing factors included age, marital status, education, and mastery. Mastery was assessed using Pearlin's personal mastery scale [36], a widely used scale with good construct validity [37] and internal reliability [38]. The mastery scale ranges from 7 to 28 where a higher score indicates better mastery.

Enabling factors included social support, place of residence, and income. Satisfaction with social support was assessed by first giving interviewees examples of positive emotional support and then asking them if they were satisfied with the amount they received (no, yes). Place of residence was either a private home, a seniors' residence, or other (rent room or subsidized housing). A seniors' residence refers to a multi-unit housing development that offers either independent living or assisted living. Residents should theoretically be either independent or have met needs, but it is possible that some residents may have developed unmet need in the years since they moved to the residence. A person was identified as having a low income using Statistics Canada's low income cut-off points for the time period of the study [39]. Five need factors were included: body mass index (BMI), self-rated health, risk for malnutrition, burden of chronic disease, and previous use of urgent health services. Self-reported height and weight were used to compute BMI. Self-rated health was dichotomized such that "excellent," "very good," and "good" responses were categorized as "good," and "fair," "poor," and "very poor" responses were categorized as "poor." Nutrition was assessed using the *Elderly Nutrition Scale *(ENS), which identifies a person as being at low, moderate, or high risk for malnutrition. The ENS has a sensitivity of 78% and 77% for identifying a person at moderate and high risk of malnutrition, respectively; an overall specificity of 77%; and good reliability [40]. Chronic disease burden was estimated using Von Korff's Chronic Disease Score (CDS) [41], a weighted index of the burden of chronic diseases based upon pharmaceutical data from administrative databases. The CDS ranges from 0 to 23, where a higher score indicates a higher burden of comorbidity. The CDS predicts both hospitalization and mortality and is positively correlated with physician ratings of disease severity (*r *= 0.57) [41]. Finally, the use of urgent health services, specifically emergency department visits and unplanned hospitalizations, in the six months prior to the baseline interview was identified from the RAMQ and MEDECHO database.

### Statistical Analysis

We report here only the analyses for women who completed the baseline interview and who gave consent for us to obtain information contained in the RAMQ and MEDECHO databases (n = 530). Descriptive statistics were computed for all study variables. Correlations between all variables were examined and a bivariate analysis of each variable with any ED visit in the 6 months following the baseline interview was conducted. Multivariable logistic regression was used to examine the effects of psychological distress and unmet need as predictors of at least one ED visit. We used SAS, version 9.1 (SAS Institute, Inc., Cary, NC) for statistical analyses. Variables having an association with ED visits with a p-value < 0.20 in the bivariate analyses or that were deemed to be clinically relevant were entered into the multivariable model. All associations with a p-value < 0.05 were retained in the final model, as were clinically relevant variables. The Akaike Information Criteria (AIC) was used to determine which variables significantly improved the predictive ability of the model and the Chi-square goodness-of-fit test was used to assess the fit of the models. The association between met and unmet need, psychological distress, and ED visits was assessed for interaction and confounding.

## Results

Descriptive analyses found that multiple ED visits were rare. Only 21 women visited the ED more than once (data not shown). Characteristics of the study participants stratified by the occurrence of at least one ED visit in the 6 months following the baseline interview are presented in Table 1. Women who visited the ED were more likely to have higher IDEPSQ-14 scores and more IADL unmet needs at the baseline interview than women who did not visit the ED. Additionally, having any PADL met need, having a low income, rating one's health as "poor", being at high risk for malnutrition, having a BMI ≥ 30 kg/m^2^, and having ED visits in the 6 months prior to the baseline were associated with a subsequent ED visit. Conversely, living in a seniors' residence (rather than a private dwelling) was associated with a reduced likelihood of visiting the ED. Correlation coefficients for IADL and PADL met and unmet needs among only disabled women ranged from -0.17 to 0.32.

**Table 1:** Characteristics of the MUNS Cohort Stratified by ED Visits within Six Months after Baseline Interview

Characteristics	No ED visit (n = 444)	Any ED visit (n = 86)	p-value
**PREDISPOSING FACTORS**			
**Age**, mean (SD) [range]	79.9 (4.0) [75-96]	79.5 (4.0) [75-89]	0.39
**Civil status**, n (%)			
Married	89 (20.1)	13 (15.1)	
Widowed	264 (59.5)	53 (61.3)	
Other	91 (20.5)	20 (23.3)	0.54
**Education**, n (%)			
Did not complete high school	190 (42.8)	44 (51.2)	
High school graduate	105 (23.7)	16 (18.6)	
Any post-secondary education	149 (33.6)	26 (30.2)	0.34
**Transformed psychological distress**,			
mean (SD) [range]	-1.9 (1.3) [(-4.6)-0.8]	-1.4 (1.2) [(-4.6)-2.7]	< 0.001
**Mastery**, mean (SD) [range]	20.8 (3.6) [9-28]	20.1 (3.8) [10-28]	0.13
**ENABLING FACTORS**			
**Dissatisfied with social support**, n (%)	67 (15.1)	14 (16.3)	0.78
**Residence**, n (%)			
Private dwelling	334 (75.2)	66 (76.7)	
Seniors' residence	75 (16.9)	6 (7.0)	
Rented room or subsidized housing	35 (7.9)	14 (16.3)	0.006
**Low income**, n (%)	156 (35.7)	46 (54.8)	0.001
Missing	7	2	
**NEED FACTORS** **Disabled in any IADL and/or PADL**	312 (70.2)	76 (88.4)	< 0.001
IADL unmet need, n (%)			
No IADL unmet needs	347 (78.1)	46 (53.5)	
1 IADL unmet need	72 (16.2)	28 (32.6)	
2+ IADL unmet needs	25 (5.6)	12 (14.0)	< 0.001
IADL met need, n (%)			
No IADL met needs	220 (49.5)	44 (51.1)	
1 IADL met need	154 (34.7)	32 (37.2)	
2+ IADL met needs	70 (15.8)	10 (11.6)	0.61
Any PADL unmet need, n (%)	35 (7.9)	12 (14.0)	0.07
Any PADL met need, n (%)	14 (3.2)	8 (9.3)	0.009
**Risk for malnutrition**, n (%)			
Low risk	177 (39.9)	19 (22.1)	
Moderate	214 (48.2)	44 (51.2)	
High risk	53 (11.9)	23 (26.7)	< 0.001
**Poor self-rated health**, n (%)	99 (22.3)	38 (44.2)	< 0.001
**Body mass index (kg/m^2^)**, n (%)			
< 25.0	256 (57.6)	22 (48.8)	
25.0 < 30.0	141 (31.8)	27 (31.4)	
30.0+	47 (10.6)	17 (19.8)	0.05
**Chronic Disease Score**, mean (SD) [range]	5.8 (5.0) 0 [1-23]	7.0 (4.9) 0 [1-23]	0.05
**Any ED visit within 6 months prior to baseline interview**, n (%)	51 (11.5)	20 (23.3)	0.003
**Any hospitalization within 6 months prior to baseline interview**, n (%)	38 (8.6)	7 (8.1)	0.90

We analyzed the associations of IADL and PADL met and unmet needs, modeled simultaneously, with ED visits without adjusting for any other variables. IADL unmet need was statistically significantly associated with an increased likelihood of visiting the ED (OR = 2.30; 95% CI: 1.60, 3.31), as was PADL met need (OR = 3.50; 95% CI: 1.26, 9.75). There was no evidence of an association between either IADL met need or PADL unmet need with ED visits.

Similarly, we analyzed the association between psychological distress scores at baseline and subsequent ED visits without adjusting for other variables. For every 1 point increase in transformed IDPESQ-14 scores at baseline, women were, on average, 40% more likely to visit the ED in the following six months (OR = 1.40; 95% CI: 1.15, 1.71).

In multivariable analysis we found that psychological distress and IADL unmet need at baseline remained statistically significant predictors of ED visits even after adjustment for other variables (Table 2). A one point increase in the transformed IDPESQ-14 score at baseline was associated with a 30% increase in the likelihood of visiting an ED. The presence of each additional IADL unmet need increased the likelihood of a woman visiting an ED by 57% (OR = 1.57; 95% CI: 1.02, 2.41). In other words, compared to women with no unmet IADL need, women with 1 IADL unmet need had a 57% (1.00 + 1.00(.57) = 1.57) increased likelihood of visiting the ED, and if they had 2 IADL unmet needs, their risk for visiting the ED increased by 57% again to 147% (1.57 + 1.57(.57) = 2.47)

**Table 2:** Multivariable Analysis of Predictors of Any ED Visit within Six Months after Baseline Interview

Variable	Odds Ratio	95% Confidence Interval	p-value
**Disability**			
IADL unmet need (0, 1, 2+)	1.57	1.02, 2.41	0.042
IADL met need (0, 1, 2+)	0.91	0.61, 1.36	0.64
PADL unmet need (0, 1+)	0.66	0.35, 1.24	0.20
PADL met need (0, 1+)	3.64	1.16, 11.4	0.027
**Risk for malnutrition**			
Low risk	Reference		
Moderate risk	1.63	0.87, 3.05	0.13
High risk	3.53	1.55, 8.01	0.003
**Body mass index**			
< 25.0 kg/m^2^	Reference		
25.0 to < 30.0 kg/m^2^	1.21	0.67, 2.20	0.53
30.0+ kg/m^2 ^	2.74	1.30, 5.79	0.008
**Chronic disease score**	1.01	0.96, 1.07	0.66
**Any ED visit six months prior to baseline**	2.16	1.14, 4.09	0.018
**Psychological distress score at baseline**	1.30	1.02, 1.67	0.038
**Mastery**	1.06	0.98, 1.15	0.15
**Low income**	1.79	1.04, 3.08	0.037
**Residence**			
Private dwelling	Reference		
Seniors' residence	0.32	0.12, 0.85	0.022
Rent room or subsidized housing	1.40	0.65, 3.04	0.39

Another facet of disability, PADL met need, was also associated with a statistically significant increase in the likelihood of visiting an ED (OR = 3.64; 95% CI 1.16, 11.4). The model was adjusted for risk for malnutrition, body mass index, previous ED visits, income, residence, chronic disease, and mastery. Von Korff's Chronic Disease Score was retained in the model despite its lack of statistical significance due to the clinical importance of chronic disease in affecting ED visits [42]. The variable mastery confounded the relationship between psychological distress and ED visits, meaning that the parameter estimate of psychological distress changed by more than 20% when mastery was included in the model compared to when mastery was not. Thus, mastery was retained in the model even though it was not statistically significant. There was no evidence of interaction between unmet need and psychological distress upon subsequent ED visits. The chi-square goodness-of-fit value for the multivariable model was 14.7 (p = 0.07), indicating that the fit of the model was acceptable. When we removed the psychological distress variable from the multivariable model, the point estimate of IADL unmet need increased to 1.75 (95% CI: 1.15, 2.66).

## Discussion

We hypothesized that unmet need but not met need would be associated with ED visits, and that the association would be stronger for PADL need than IADL need. Our findings were only partially supportive of this hypothesis. As we expected, both IADL unmet need and elevated psychological distress at the baseline interview were predictors of visiting the ED at least once in the subsequent six months after adjustment for risk for malnutrition, body mass index, chronic disease score, previous ED visits, mastery, low income, and residence (Table 2). Contrary to our hypothesis, however, PADL unmet need did not predict ED visits yet PADL met need did.

The finding of a link between IADL unmet need and subsequent ED visits is not surprising. Unmet need is associated with negative physical consequences such as being unable to follow a special diet or take medications as directed [3,11], which can lead to a health emergency. The link between psychological distress and ED visits, however, is less clear. If it possible that psychological distress may have negative effects on physical health leading to the use of health services, or that distressed people are more likely to seek health care, possibly because of somatization of psychological distress leading to physical health complaints for which a person seeks medical attention [43]. Alternatively, seniors with unmet need may feel socially isolated and lonely, and may seek comfort and attention within the health care system [44].

Unlike unmet IADL need, we found no evidence that unmet PADL need predicted ED visits in women. Although the association was not statistically significant, the fact that the point estimated suggested a protective effect was unexpected (OR = 0.66; 95% CI: 0.35, 1.24). A likely explanation is that the point estimate is unstable because so few study participants had PADL unmet need. It is possible that the addition of a few more people with PADL unmet need could markedly alter the point estimate, or even reverse the direction of the association. Another unexpected finding was that, compared to women with no PADL need, women with *met *PADL need were over three times more likely to visit an ED. A possible explanation for this finding may be that an ED is more accessible for women whose needs are met than it is for those who do not receive assistance. For example, family members or homecare workers who provide assistance to a disabled elderly person may also monitor the health of the latter and identify important health problems requiring medical attention that might otherwise go unnoticed or ignored. Moreover, he or she can also provide the individual with transportation to an ED. Whether women with PADL unmet need are less likely to have or recognize a health emergency or be able to visit the ED remains to be determined in future research.

We also hypothesized that psychological distress acted as either a confounder and/or effect modifier in the association between unmet need and ED visits, and our results confirmed this. When psychological distress was excluded from statistical modeling, the odds ratio for IADL unmet need increased from 1.57 (95% CI: 1.02, 2.41) to 1.75 (95% CI: 1.15, 2.66), suggesting that failing to adjust for psychological distress may overestimate the magnitude of the association between IADL unmet need and subsequent ED visits in elderly women.

Most of the covariates in our analyses are health-related and have a direct association with ED visits. However, mastery and residence do not have an obvious connection with the use of health services yet were both statistically significant in our multivariable model. We found that mastery confounded the association between psychological distress and ED visits, such that failing to include it in the model underestimated the magnitude of the association between psychological distress and ED visits. Mastery is the extent to which a person feels in control over the environment [36] and has been shown to increase psychological resilience and facilitate adaptation to some of life's most stressful events, including health problems and functional decline [45,46]. We also found that women who lived in a seniors' residence were statistically significantly less likely to visit the ED than women living in a private home. Why this might be so is not known, but we believe that women living in a seniors' residence may benefit from access to health care professionals who staff the residence or scheduled activity programs promoting exercise.

Our findings for mastery and residence suggest a possible solution for situations where home care services are not available or are not desired by an elderly person. Adult day support programs provide out-of-home services to elderly citizens with varying levels of ability. These programs are where seniors can learn adaptive strategies or acquire adaptive devices to address their unmet needs, and which may in turn improve their sense of mastery. Other advantages of attending day programs, similar to the advantages of living in a seniors' residence, include access to medical professionals, access to exercise and rehabilitation programs, and increased socialization with peers [47,48]. Thus, day programs should be evaluated to determine their feasibility and cost-effectiveness with respect to meeting at least some of the needs of community-dwelling disabled elderly persons.

Strengths of this research include its prospective study design, population-based sample, the linkage of questionnaire data with administrative databases to provide accurate information on health service utilization, and the implementation of in-depth, face-to-face interviews which provided high-quality data.

Limitations of the study include the possible introduction of selection and misclassification biases, and the possibility of confounding that may distort the true associations between variables of interest. Volunteer bias, a form of selection bias, may be present if individuals who were more severely disabled (and thus had more unmet needs) *and *had more severe psychological distress were less likely to agree to participate in the MUNS than their healthier and less distressed peers. This would make it more difficult to identify the association of these factors with ED visits and it is likely that the true association is greater than the association we observed.

Misclassification of the various components of disability (i.e., met need and unmet need) may have occurred as a result of having the participants self-report their physical ability. It can be argued that a more accurate measurement of disability is obtained when a physical therapist evaluates an individual's ability to perform an ADL. However, if a person reports being unable to perform a task even when she is physically capable of doing so, she typically will not perform the task and hence *does *have a need. Thus, self-report was the preferred way to assess disability and unmet need in this research.

An additional challenge of this research was to address the potential for confounding by disability, since more severe disability is associated with both an increased occurrence of unmet need [49] and more severe psychological distress [50]. Although we adjusted for disability, residual confounding may still be present as a result of collapsing categories of unmet and met need: participants in the "two or more" needs category may have had three IADL needs and those in the "one or more" needs category may have had up to six PADL needs. This would make them more disabled than indicated by our categorization. However, only 30 (5.2%) and 16 (2.8%) women were disabled in a greater number of IADL and PADL, respectively, than were indicated by the collapsed categories, and these numbers are insufficient to substantially confound the results.

## Conclusions

In sum, both unmet needs and psychological distress can cause hardship and suffering, may accelerate the decline of health and functional ability, and can lead to an increased use of emergency health services. It is essential to provide elderly persons with the support they need - and will accept - to adapt, both physically and mentally, to declining health and function. Furthermore, future research on unmet need and health services use should include measures of psychological distress to control for confounding and improve the predictive ability of statistical models.

## Competing interests

The authors declare that they have no competing interests.

## Authors' contributions

JQ developed the study concept of secondary analysis within Montreal Unmet Needs Study, analyzed and interpreted the data, and prepared the manuscript. CW developed the study concept and design of Montreal Unmet Needs Study, acquired study participants and data, acquired funding, and assisted with the interpretation of data and preparation of the manuscript. AL assisted with interpretation of data and preparation of the manuscript. All authors read and approved the final manuscript.

## Pre-publication history

The pre-publication history for this paper can be accessed here:

http://www.biomedcentral.com/1471-2318/11/86/prepub
